# Pre-Bed Casein Protein Supplementation Does Not Enhance Acute Functional Recovery in Physically Active Males and Females When Exercise is Performed in the Morning

**DOI:** 10.3390/sports7010005

**Published:** 2018-12-28

**Authors:** Eva Apweiler, David Wallace, Sarah Stansfield, Dean M. Allerton, Meghan A. Brown, Emma J. Stevenson, Tom Clifford

**Affiliations:** 1 Faculty of Health Medicine and Life Sciences, Maastricht University, 6211 LK Maastricht, The Netherlands; evaapweiler@gmail.com (E.A.); david.wallace@gmx.de (D.W.); 2 School of Biomedical Sciences, Newcastle University, Newcastle NE2 4HH, UK; sarah.stansfield@newcastle.ac.uk (S.S.); dean.allerton@newcastle.ac.uk (D.M.A.); 3 School of Sport and Exercise, University of Gloucestershire, Gloucester GL2 9HW, UK; mbrown15@glos.ac.uk; 4 Human Nutrition Research Centre, Institute of Cellular Medicine, Newcastle University, Newcastle NE2 4HH, UK; emma.stevenson@newcastle.ac.uk

**Keywords:** strength, muscle soreness, nutrition, supplement, exercise recovery

## Abstract

This study examined whether consuming casein protein (CP) pre-sleep could accelerate acute recovery following muscle-damaging exercise. Thirty-nine active males and females performed 100 drop jumps in the morning, consumed their habitual diet during the day, and then within 30 min pre-bed consumed either ~40 g of CP (*n* = 19) or ~40 g of a carbohydrate-only control (CON) (*n* = 20). Maximal isometric voluntary contractions (MIVC), countermovement jumps (CMJ), pressure-pain threshold (PPT), subjective muscle soreness and the brief assessment of mood adapted (BAM+) were measured pre, 24 and 48 h following the drop jumps. MIVC decreased in CP and CON post-exercise, peaking at 24 h post (CP: −8.5 ± 3.5 vs. CON: −13.0 ± 2.9%, respectively); however, no between-group differences were observed (*p* = 0.486; η_p_^2^ =0.02). There were also no group differences in the recovery of CMJ height, PPT and BAM+ (*p* > 0.05). Subjective muscle soreness increased post-exercise, but no group differences were present at 24 h (CP: 92 ± 31 mm vs. CON: 90 ± 46 mm) or 48 h (CP: 90 ± 44 mm vs. CON: 80 ± 58 mm) (*p* > 0.05). These data suggest that pre-bed supplementation with ~40 g of CP is no more beneficial than CON for accelerating the recovery following muscle-damaging exercise.

## 1. Introduction

Unaccustomed and strenuous contractile activity can damage the proteins in muscle and connective tissues [1,2]. Such damage results in inflammation, swelling, muscle soreness, and a loss of force-generating capacity [1]. Because these symptoms can persist for several days before fully recovering, physical activity in the ensuing days might be compromised [1]. This is especially problematic in athletes who need to train almost daily to develop the requisite technical and physical abilities for their sport. However, recreationally active and novice exercisers may also be affected, as tasks required for daily living such as stair climbing and walking can also be impaired in the days following muscle-damaging exercise [3].

In an attempt to accelerate the recovery processes and alleviate the deleterious effects of exercise-induced muscle damage (EIMD), athletes and recreational exercisers alike often supplement with nutritional products. The most popular recovery supplements tend to be protein-based [4]. The rationale is that protein ingestion stimulates muscle protein synthesis (MPS) and reduces muscle protein breakdown (MPB) effects, which, at least in theory, help to accelerate myofibrilar re-conditioning following a damaging insult [4,5,6]. While individual studies have come to equivocal conclusions, a recent meta-analysis concluded that whey protein supplementation had small to moderate benefits for the recovery of muscle function [7]. Thus, the general consensus is that protein supplementation is likely to facilitate recovery following muscle-damaging exercise. 

Because the benefits of post-exercise protein supplementation are linked to increased MPS, it would be reasonable to assume that maximising this response in the 24 h following muscle-damaging exercise would help to expedite the recovery process. During the day, MPS can be maximised by consuming 0.4 g·kg^−1^ of a leucine rich protein source every 2–3 h or 3–4 times per day [8]. However, this pattern of eating does not appear to influence overnight MPS, which tends to be low even when 20–30 g of protein is consumed late in the evening (~20:00–22:00) [9,10,11]. To potentiate overnight MPS, an additional bolus of protein appears to be required closer to sleep. Indeed, in a series of recent studies, it has been shown that ingesting a large bolus (~40 g) of slowly digestible casein protein (CP) within 30 min of going to sleep (~23:30) increases overnight MPS and whole body net protein balance [9,12,13,14]. Indeed, some of these studies showed that overnight MPS rates are 22–33% higher when ~40 g of CP compared to an isocaloric carbohydrate drink is consumed 30 min prior to sleep [9,10]. These findings suggest that pre-sleep CP intake might be a strategic opportunity to further augment myofibrilar re-synthesis over a 24 h period. 

Two recent studies have exploited the effects of pre-sleep protein ingestion for its potential to ameliorate EIMD when exercise is performed late in the evening. West et al. [15] showed that ingesting 25 g of whey protein pre-sleep accelerated the recovery of strength and power 24 h after a bout of evening resistance exercise (19:00). Similarly, we showed that ingesting ~40 g of CP pre-sleep attenuated muscle soreness and enhanced the recovery of muscle function in the 60 h following an evening soccer match (19:00) [16]. These studies suggest that pre-sleep protein ingestion might facilitate recovery when exercise is performed in the evening. However, whether such effects are observed when exercise is performed in the morning—and there are plenty of opportunities for regular protein servings before sleep—is unknown. Because MPS is potentiated by prior resistance exercise, pre-sleep CP ingestion appears to have greater effects on overnight MPS and myofibrilar re-conditioning when exercise is performed later in the evening [12,13]. Yet, theoretically, stimulating overnight MPS would increase the overall MPS response in the 24 h following the exercise bout and, thus, if total protein intake throughout the course of a day is the major determinant of muscle protein accretion—as suggested by many [17,18]—such a strategy could still augment muscle protein turnover and possibly functional recovery. Thus, the aim of the present study was to examine whether consuming an additional bolus of casein protein (CP) pre-sleep could accelerate acute functional recovery following muscle-damaging exercise performed in the morning. We hypothesised that muscle soreness and muscle function deficits would be attenuated by pre-sleep CP ingestion. 

## 2. Methods

### 2.1. Participants

Forty healthy physically active males and females (defined as performing moderate intensity exercise ≥2 days per week) provided written informed consent to participate in this study (see Table 1 for physical characteristics). One of the participants did not consume their entire supplement and therefore was excluded from the data analysis; thus, final data analysis was performed on 39 participants (19 females and 20 males). Participants underwent a health screening and were excluded if they did not agree to avoid using any dietary supplements (i.e., multivitamins, whey protein, and creatine), pain relief medications (i.e., nonsteroidal anti-inflammatory drugs), or putative recovery treatments (i.e., compressions garments, massage) throughout testing. To control for the potential influence of different menstrual cycle phases on EIMD, we asked all female participants to complete a menstrual cycle questionnaire at familiarisation [19,20] and used this to schedule testing during the same monthly phase (follicular). There is some suggestion that males and females respond differently to muscle damaging exercise and possibly even protein ingestion [21]; therefore, we also performed a sub-group analysis of the data for males and females separately to test for any sex-specific effects. The study procedures received ethical approval from Newcastle University (14733882). 

### 2.2. Experimental Protocol 

The study used a randomised, double blind, placebo-controlled, independent groups design. Following familiarisation, in which height (cm), body mass (kg), maximum isometric voluntary contraction (MIVC) and counter movement jump height (CMJ) were collected, participants were randomly allocated into a casein protein (CP) or carbohydrate-only control (CON) group. The participant groups were matched on this initial MIVC score. Within seven days of the familiarisation, participants visited the laboratory for three consecutive testing days (between 07:30 and 09:00). On day 1, participants arrived fasted and baseline values for muscle soreness, pressure pain threshold, CMJ, MIVC and subjective wellbeing were recorded. They then completed 100 drop jumps, designed to induce muscle damage. Following this, participants consumed a whey protein supplement (see Table 2 for nutritional composition) and were provided with either a CP or CON supplement to be consumed before bed (10:00–11:30). The participants returned to the lab 24 and 48 h following the jumps to repeat the dependent variables. 

### 2.3. Muscle-Damaging Protocol

To induce muscle damage, participants performed 100 drop jumps (4 sets of 25, separated by 2 min of passive recovery) from a 60 cm high box. For the jumps, they dropped off the box and then immediately performed a vertical jump. Participants were instructed to descend to 90° knee flexion for each jump and received verbal cues to ensure the vertical jump was performed with maximal effort (e.g., they jumped as high as possible) and correct technique was maintained throughout. The jumps were performed every 10 seconds. We have previously found this protocol to be a reliable method of inducing muscle damage in a young, active population [22,23]. 

### 2.4. Muscle Soreness 

Subjective muscle soreness was assessed with a visual analogue scale (VAS). Participants descended to 90° of knee flexion and rated the level of pain in their lower limbs on a 200 mm scale anchored by the terms “no soreness” and “unbearably painful” on each end. 

### 2.5. Pressure Pain Threshold 

PPT was measured with a handheld algometer (Wagner Instruments, Greenwich, Conn., USA) and with the participant lying supine. As in several previous studies [22,23], PPT was recorded at the following three sites: vastus lateralis (mid-way between the superior aspect of the greater trochanter and head of the tibia), rectus femoris (mid-way between the anterior patella and inguinal fold), and gastrocnemius (medial aspect of the calf at relaxed maximum girth). PPT was measured by applying constant pressure to each of these sites until the participant indicated that they felt pain. This was done twice, unless the values differed by more than 10 N, then a third recording was taken, and the average of the two closest values was used for data analysis. The inter-day coefficient of variance (CV) for this protocol was calculated as <12%.

### 2.6. Counter Movement Jump

An Opto-jump system was used to measure CMJ performance (Optojump Next, Bolzano, Italy). This procedure has been described in detail elsewhere [22,23]. Briefly, with hands on hips, participants descended into a squat position before jumping vertically with maximum effort. Three efforts were performed; each was separated by a 60 s recovery period. The peak value was used for analysis. The CV for this procedure was <2.5%.

### 2.7. Maximum Voluntary Isometric Contraction

As described previously [22], MIVC was measured using a portable strain gauge (MIE Medical Research Ltd., Leeds, UK). In a seated position at a 90° knee angle participants pushed with maximal force against a perspex gauze attached to their right ankle just above the malleoli. The three attempts were separated by 60 seconds of recovery; the peak value was used for analysis. The CV for this procedure has been calculated as <4% in our laboratory. 

### 2.8. Supplementation

The nutritional composition of each supplement is provided in Table 2. Following the drop jumps, each participant consumed the same whey protein + carbohydrate drink (Science in Sport; REGO Rapid Recovery; chocolate flavour). This drink was provided to all participants to standardize post-exercise protein intake [24] and reflect real-world practices, which recommend a fast absorbing protein be consumed within 60 min of exercise cessation (Aragon et & Schoenfeld, 2013). Participants were also given either a CP drink (Micellar casein protein, Myprotein, UK) or a CON (Maltodextrin, Myprotein, UK) with instructions to consume this with ~350 ml of water within 30 min before bed. We chose a CP dose of ~40 g, as this appears to the most effective dose for maximally stimulating overnight MPS [10,14]. Both supplements were flavour matched by adding 7.5 g of a low-calorie chocolate powder (Cadburys Highlights, Hershey, PA, USA). The drinks were prepared in identical 500 mL bottles by an individual not involved in data collection. 

### 2.9. Performance Readiness

Performance readiness was assessed with the Brief Assessment of Mood adapted (BAM+) developed by Shearer et al. [25]. The BAM+ is comprised of 10 questions relating to individuals subjective wellbeing and has been described in detail elsewhere [25]. Example questions include ‘How well do you feel you’ve slept?’ and ‘how confident do you feel?’. Each question was answered by marking a line on a 100 mm VAS anchored by ‘not at all’ and ‘extremely well’ at each end. These lines were measured with a ruler and an overall score calculated. 

### 2.10. Dietary and Exercise Control

Participants abstained from exercise from 48 h prior to the exercise protocol until the end of the testing period. Participants did not alter their habitual intakes but were required to keep a 3-day food diary (Intake24, Newcastle University, UK). 

## 3. Data Analysis 

All data are expressed as mean ± standard deviation (SD) and statistical significance was set at *p* < 0.05 prior to analyses. MIVC, CMJ, muscle soreness (MS), PPT and BAM+ values were analysed using a mixed model analysis of variance (ANOVA) with two treatment levels (CP vs. CON) and three repeated measures time points (before exercise; PRE, 24 h and 48 h post-exercise). Separate ANOVAs were performed for male subjects, female subjects, and all subjects (both males and females). Another ANOVA was performed to examine any differences in the dependent variables in males vs. females, irrespective of supplementation. CMJ, PPT for males-only analysis and VAS and PPT for females-only analysis were not normally distributed (based on *p* < 0.05 on the Kolmogorov–Smirnov test) and therefore log transformed for analysis. Least significance difference (LSD) post hoc tests were performed if a main time, interaction or group effect was observed (*p* ≤ 0.05). If sphericity was violated, Greenhouse–Geisser adjustments were used. Multiple Student’s t-tests were used to analyse group differences in physical characteristics and dietary intakes. All data were analysed using IBM SPSS Statistics 23 for Windows (Surrey, UK). Partial-eta^2^ (η_p_^2^) effect size statistics are presented and were considered either small (0.01–0.06), medium (0.06–0.14) or large (≥0.14) changes. 

## 4. Results

There were no between-group differences in participants physical characteristics (*p* > 0.05) and macronutrient intakes (energy, carbohydrate, protein and fat) the day before and the day following the exercise test (data not shown; *p* > 0.05). In the 24 h following exercise, there were no differences in habitual intakes between the two groups (this includes the post-exercise protein drink that all participants consumed; *p* > 0.05; Table 1). However, as expected, with the pre-sleep interventions, protein intake was greater and carbohydrate intake lower in the CP group (*p* < 0.05; Table 1). 

### 4.1. All Participants 

After exercise, the magnitude of muscle damage, as indicated by changes in the dependent variables, did not differ between the males and females (*n* = 20 vs. *n* = 19, respectively), suggesting that sex did not have a significant influence on the functional markers of exercise recovery (interaction effect; *p* > 0.05). 

**Figure 1:**
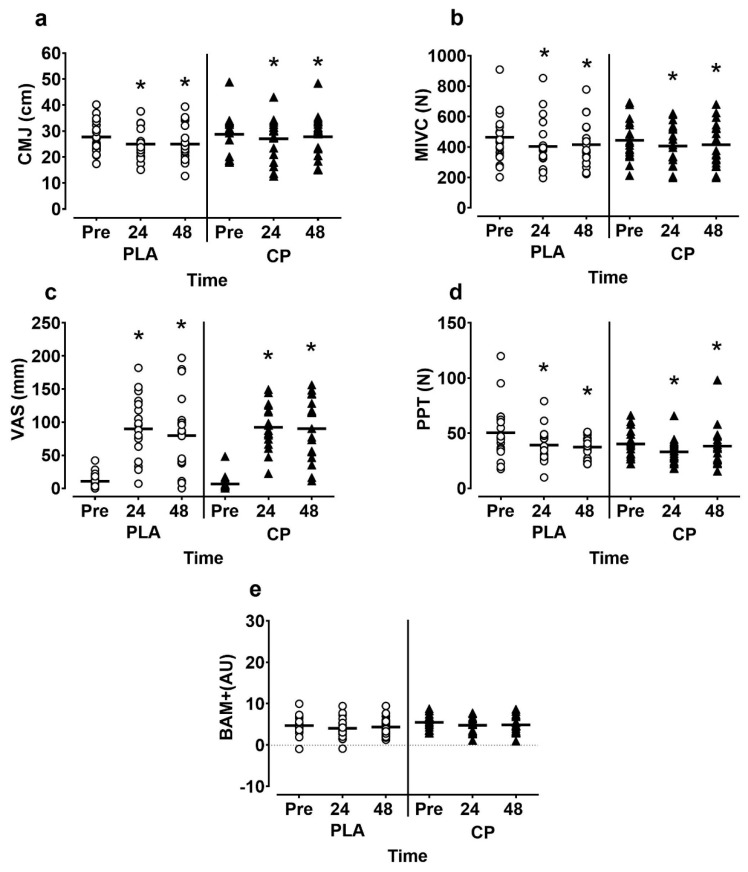
and (**a**) Countermovement jump (CMJ) height; (**b**) **maximal isometric voluntary contractions (MIVC)**; (**c**) **subjective muscle soreness (VAS)**; (**d**) pressure pain threshold (PPT); (**e**) **Brief assessment of mood adapted (BAM+)** in all participants pre, 24 and 48 h following muscle damaging exercise. CP, casein protein; CON, carbohydrate-only control. * denotes significant time effect (*p* < 0.05).

Data for all participants for analysis of CON vs. CP is presented in Figure 1. Subjective muscle soreness was increased and PPT decreased post-exercise (time effects; *p* < 0.05) but no interaction effects were present for either measure (*p* > 0.05). There was a time effect for CMJ (*p* = 0.001; η_p_^2^ = 0.20) which was reduced by 5–10% 24 h post-exercise. No interaction effects for CMJ were observed (*p* = 0.211; η_p_^2^ = 0.042). Similarly, MIVC decreased post-exercise (time effect; *p* = 0.001; η_p_^2^ = 0.24). At 24 h post, MIVC was reduced by 37 N in CP and 61 N in CON; no interaction effects were present (*p* = 0.486; η_p_^2^ = 0.02). There were no time or interaction effects for BAM+ (*p* > 0.05).

### 4.2. Females

**Figure 2:**
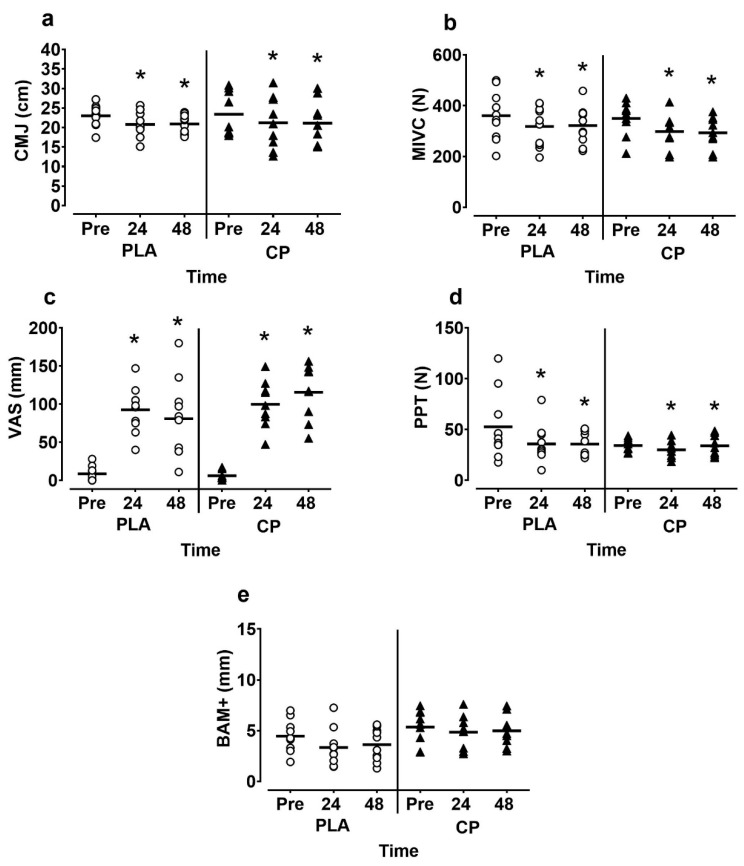
(**a**) Countermovement jump (CMJ) height; (**b**) maximal isometric voluntary contractions (MIVC); (**c**) subjective muscle soreness (VAS); (**d**) pressure pain threshold (PPT); (**e**) Brief assessment of mood adapted (BAM+) in female participants pre, 24 and 48 h following muscle damaging exercise. CP, casein protein; CON, carbohydrate-only control. * denotes significant time effect (*p* < 0.05).

Data for female participants is presented in Figure 2. For females, muscle soreness increased and PPT decreased post-exercise (time effect; *p* > 0.05). Muscle soreness peaked at 24 h in CON (92.5 ± 30.0 mm) and at 48 h in CP (115.5 ± 35.8 mm). No interaction effects were observed for muscle soreness or PPT (*p* = 0.456; η_p_^2^ = 0.37 and *p* = 0.21; η_p_^2^ = 0.09, respectively). MIVC was reduced by 10–15% 24 and 48 h post-exercise (time effect; *p* = 0.001; η_p_^2^ = 0.47) but there were no between-group differences (interaction effect; *p* = 0.683; η_p_^2^ = 0.02). CMJ performance was similarly decreased post-exercise (*p* = 0.001; η_p_^2^ = 0.441) and there was no group interaction effect (*p* = 0.966; η_p_^2^ = 0.02). BAM+ scores tended to be lower post-exercise (*p* = 0.064; η_p_^2^ = 0.150) but there were no interaction effects (*p* = 0.655; η_p_^2^ = 0.02). 

### 4.3. Males

**Figure 3:**
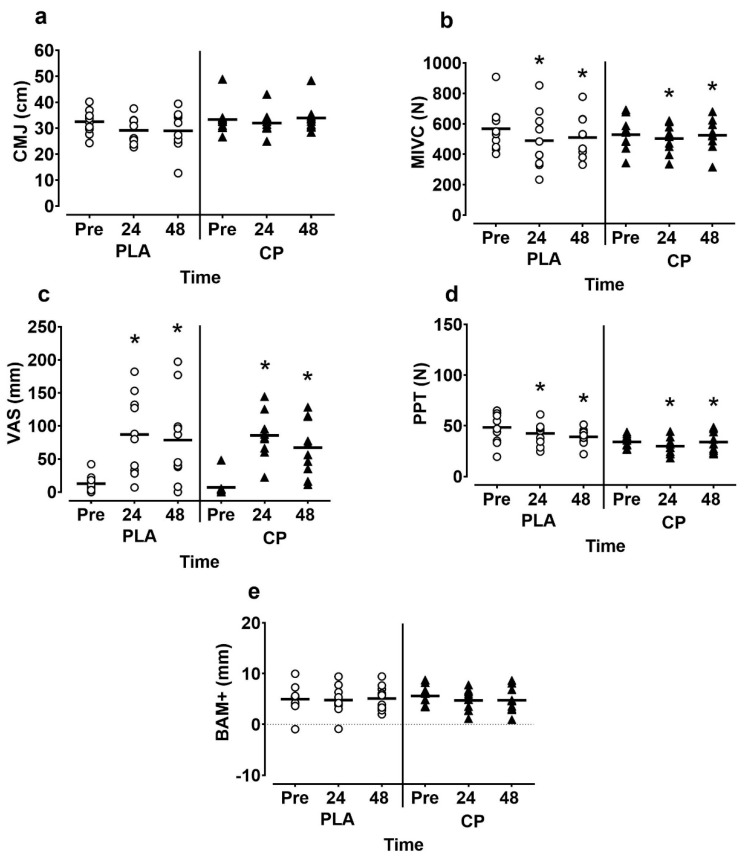
(**a**) Countermovement jump (CMJ) height; (**b**) maximal isometric voluntary contractions (MIVC); (**c**) subjective muscle soreness (VAS); (**d**) pressure pain threshold (PPT); (**e**) Brief assessment of mood adapted (BAM+) in male participants pre, 24 and 48 h following muscle damaging exercise. CP, casein protein; CON, carbohydrate-only control. * denotes significant time effect (*p* < 0.05).

Data for male participants is presented in Figure 3. Muscle soreness increased and PPT decreased in both groups at 24 and 48 h post-exercise (time effect; *p* > 0.05); however, no interaction effects were observed for either subjective soreness (*p* = 0.854; η_p_^2^ = 0.07) or PPT (*p* = 0.316; η_p_^2^ = 0.06). CMJ height did not fall below baseline levels at any time point post-exercise (time effect; *p* = 0.143; η_p_^2^ = 0.10) and no interaction effects were present (*p* = 0.142; η_p_^2^ = 0.10). At 24 post-exercise CMJ height was −9.4 ± 14.7% in the CON group and −3.7 ± 4.0 in the CP group. MIVC decreased post-exercise (time effect; *p* = 0.02; η_p_^2^ = 0.18) but there were no between-group differences at any time point (*p* = 0.226; η_p_^2^ = 0.07). The BAM+ showed no time (*p* = 0.532; η_p_^2^ = 0.29) or interaction effects (*p* = 0.585; η_p_^2^ = 0.02). 

## 5. Discussion 

A number of previous studies have reported that ingesting ~40 g of CP within 30 min of sleep augments overnight MPS rates, resulting in a more positive net protein balance and the formation of new myofibrilar proteins during sleep (reviewed by [26]). Recent studies suggested that these effects might translate to improved functional recovery in the days following exercise performed in the evening e.g., closer to sleep [15,16]. The present study aimed to extend upon these findings and assess whether CP ingestion might attenuate muscle soreness and decrements in muscle function when exercise is performed in the morning and followed by a normal pattern of eating with sufficient protein intake. Contrary to our hypothesis, ingesting a large bolus of CP pre-sleep did not affect functional recovery in the 48 h following a strenuous bout of eccentrically-biased exercise performed in the morning, irrespective of sex. 

These findings are in contrast to our recent study in professional soccer players where we found that ingesting ~40 g of CP before sleep accelerated the recovery of CMJ and reactive strength performance in the 60 h following an evening soccer match [16]. They are also not in agreement with the findings of West et al. [15] who reported muscle strength and power recovered quicker 24 h following a bout of resistance exercise when 25 g of whey protein (vs. an isocaloric control) was consumed pre-sleep. While the discrepancy between these studies and the present study could be linked to the different training status of the subjects or the exercise modes used, a more likely explanation relates to the timing of the exercise. It was recently shown that overnight myofibrilar re-synthesis following pre-sleep CP ingestion is further augmented by prior resistance exercise [12,13], ostensibly due to the independent effects of contractile activity on MPS—which, although can remain elevated for up to 48 h, are still attenuated ≥3 h following an exercise bout [8,27]. This suggests that overnight myofibrilar re-conditioning is probably greater when exercise is performed later in the evening. As such, the recovery benefits of pre-sleep protein ingestion observed in previous work could be due to the potentiating effect of the evening exercise bouts on MPS. In contrast, because exercise was performed in the morning in the present study, the post-exercise anabolic response or heightened sensitivity to amino acid ingestion could have been sufficiently dissipated when the CP was ingested to have little or at least a smaller additive benefit [8]. We did not measure muscle protein turnover in the present study and can therefore only speculate on these effects. However, there is some evidence to support the contention that augmenting overnight MPS facilitates the recovery of acute muscle function. Indeed, West et al. [15] showed concurrent improvements in net protein balance and strength recovery in the 24 h following a bout of resistance exercise. Taking these and the present findings into consideration, perhaps pre-sleep protein ingestion represents a useful strategy for accelerating the recovery of muscle function after evening exercise bouts but not when performed in the morning. 

A recent study showed that 35 g of CP consumed either during the day or pre-sleep led to similar improvements in muscle strength and size when combined with 12 weeks of progressive resistance training [18]. Protein intakes were matched in this study, and so the authors concluded that total protein intake over the 24 h period following exercise is more important than the timing of intake when it comes to muscular adaptations. Nonetheless, in the present study, total daily protein intake was greater in the intervention group—similar to previous studies showing benefits of pre-sleep CP on muscle protein metabolism [9,10,12,14]—and we still did not observe any positive effects. This could be because the subject’s habitual protein intake in the 24 h following the exercise bout were already sufficient to maximise myofibrilar re-conditioning and therefore the additional 40 g intake of CP had no further benefits—at least for acute functional recovery. Indeed, previous studies have come to the conclusion that daily protein intakes of 1.2–1.6 g∙kg^−1^ are likely sufficient to maximise muscle protein turnover and subsequent adaptation [8]. Given that the subjects in both groups ingested ≥1.5 g∙kg^−1^ of protein in the 24 h following exercise (Table 1), the additional protein from the pre-sleep CP might have offered little further benefit. It is important to note, however, that the aforementioned guidelines are generally aimed at those looking to maximise chronic muscular adaptations such as hypertrophy. We are unaware of any specific guidelines or studies that have assessed, in a dose response manner, the optimal protein intakes for attenuating EIMD. This is an area of research that should be explored in the future. 

The main limitation of this study is that we did not measure MPS or whole-body protein turnover. We therefore cannot confirm whether the CP intervention did in fact increase overnight MPS. However, given the extensive number of studies that have now demonstrated these effects, we feel confident that the intervention would have positively affected overnight MPS rates at least to some extent. We also think our focus on muscle function markers over MPS measures is a strength of the study, given they have the most practical relevance for athletic populations and are still the most valid markers of EIMD [28]. A second potential limitation of this study is that we did not control dietary intake or, more specifically, directly match protein intakes in the two groups. Although the food diaries indicated that the macronutrient composition between the CP and CON groups did not differ, the inherent limitations of collecting and analysing food diaries means that we cannot rule out the presence of subtle differences that could have affected the results. 

In conclusion, this is the first study to assess the effects of pre-sleep CP ingestion on indirect markers of EIMD in recreationally active males and females following exercise performed in the morning. We saw no differences in the recovery markers between males and females and found that the pre-sleep CP ingestion was no more effective than consuming a carbohydrate-only control for acute functional recovery. From a practical perspective, our findings suggest that protein intakes of ~1.5 g∙kg^−1^ in the 24 h following muscle-damaging exercise performed in the morning are sufficient to maximise acute functional recovery, and that no further benefits can be gleaned by consuming an additional bolus of pre-sleep CP. 

## Figures and Tables

**Table 1:** Participants’ physical characteristics and macronutrient intake in the 24 h following exercise.

Characteristics and Dietary Intake	CP (*n* = 19)	CON (*n* = 20)
Age (years)	23 ± 2	24 ± 3
Sex	10 males, 9 females	10 males, 10 females
Body mass (kg)	67.9 ± 11.2	69.9 ± 12.8
Height (cm)	1.71 ± 0.81	1.73 ± 0.96
Daily energy intake (kcal∙kg^−1^) *	28.70 ± 7.19 [31.89 ± 7.09]	32.68 ± 11.13 [35.41 ± 11.29]
Carbohydrate (g∙kg^−1^) *	3.69 ± 0.95 [3.72 ± 0.95] #	4.09 ± 1.53 [4.72 ± 1.56]
Protein (g∙kg^−1^) *	1.50 ± 0.52 [2.12 ± 0.51] #	1.58 ± 0.59 [1.60 ± 0.59]
Fat (g∙kg^−1^) *	1.00 ± 0.46 [1.04 ± 0.46]	1.19 ± 0.51 [1.20 ± 0.51]

* Values in brackets represents intake with pre-sleep casein protein (CP) or carbohydrate-only control (CON) supplement. # denotes group difference with supplementation (*p* < 0.05).

**Table 2:** Macronutrient composition of supplements.

Supplement	PRO + CHO *	CP	CON
Energy (Kcal)	271.0	210.0	184.0
Carbohydrate (g)	38.0	4.9	42.8
Protein (g)	24.0	41.2	1.3
Fat (g)	2.6	2.9	0.8

* Recovery drink consumed by all participants immediately post-exercise. CP, casein protein; CON, carbohydrate-only control.

## References

[1] Hyldahl R.D., Hubal M.J. (2014). Lengthening our perspective: Morphological, cellular, and molecular responses to eccentric exercise. Muscle Nerve.

[2] Paulsen G., Ramer Mikkelsen U., Raastad T., Peake J.M. (2012). Leucocytes, cytokines and satellite cells: What role do they play in muscle damage and regeneration following eccentric exercise?. Exerc. Immunol. Rev..

[3] Dannecker E.A., Knoll V., Robinson M.E. (2008). Sex differences in muscle pain: Self-care behaviors and effects on daily activities. J. Pain.

[4] Pasiakos S.M., Lieberman H.R., McLellan T.M. (2014). Effects of protein supplements on muscle damage, soreness and recovery of muscle function and physical performance: A systematic review. Sports Med..

[5] Farup J., Rahbek S.K., Knudsen I.S., de Paoli F., Mackey A.L., Vissing K. (2014). Whey protein supplementation accelerates satellite cell proliferation during recovery from eccentric exercise. Amino Acids.

[6] Rowlands D.S., Nelson A.R., Raymond F., Metairon S., Mansourian R., Clarke J., Stellingwerff T., Phillips S.M. (2015). Protein-leucine ingestion activates a regenerative inflammo-myogenic transcriptome in skeletal muscle following intense endurance exercise. Physiol. Genom..

[7] Davies R.W., Carson B.P., Jakeman P.M. (2018). The effect of whey protein supplementation on the temporal recovery of muscle function following resistance training: A systematic review and meta-analysis. Nutrients.

[8] Phillips S.M., Chevalier S., Leidy H.J. (2016). Protein “requirements” beyond the RDA: Implications for optimizing health. Appl. Physiol. Nutr. Metab..

[9] Res P.T., Groen B., Pennings B., Beelen M., Wallis G.A., Gijsen A.P., Senden J.M.G., Van Loon L.J. (2012). Protein ingestion before sleep improves postexercise overnight recovery. Med. Sci. Sports Exerc..

[10] Kouw I.W., Holwerda A.M., Trommelen J., Kramer I.F., Bastiaanse J., Halson S.L., Wodzig W.K.W.H., Verdijk L.B., van Loon L.J. (2017). Protein ingestion before sleep increases overnight muscle protein synthesis rates in healthy older men: A randomized controlled trial. J. Nutr..

[11] Beelen M., Tieland M., Gijsen A.P., Vandereyt H., Kies A.K., Kuipers H., Saris W.H.M., Koopman R., van Loon L.J. (2008). Coingestion of carbohydrate and protein hydrolysate stimulates muscle protein synthesis during exercise in young men, with no further increase during subsequent overnight recovery. J. Nutr..

[12] Holwerda A.M., Kouw I.W., Trommelen J., Halson S.L., Wodzig W.K., Verdijk L.B., van Loon L.J. (2016). Physical Activity Performed in the Evening Increases the Overnight Muscle Protein Synthetic Response to Presleep Protein Ingestion in Older Men. J. Nutr..

[13] Trommelen J., Van Loon L.J. (2016). Pre-sleep protein ingestion to improve the skeletal muscle adaptive response to exercise training. Nutrients.

[14] Trommelen J., Kouw I.W., Holwerda A.M., Snijders T., Halson S.L., Rollo I., Verdijk L.B., van Loon L.J. (2017). Presleep dietary protein-derived amino acids are incorporated in myofibrillar protein during postexercise overnight recovery. Am. J. Physiol.-Endocrinol. Metab..

[15] West D.W., Abou Sawan S., Mazzulla M., Williamson E., Moore D.R. (2017). Whey protein supplementation enhances whole body protein metabolism and performance recovery after resistance exercise: A double-blind crossover study. Nutrients.

[16] Abbott W., Brett A., Cockburn E., Clifford T. (2018). Pre-sleep casein protein ingestion accelerates functional recovery in professional soccer players. Int. J. Sports Physiol. Perform..

[17] Aragon A.A., Schoenfeld B.J. (2013). Nutrient timing revisited: Is there a post-exercise anabolic window?. J. Int. Soc. Sports Nutr..

[18] Joy J.M., Vogel R.M., Broughton K.S., Kudla U., Kerr N.Y., Davison J.M., Wildman R.E.C., DiMarco N.M. (2018). Daytime and nighttime casein supplements similarly increase muscle size and strength in response to resistance training earlier in the day: A preliminary investigation?. J. Int. Soc. Sport. Nutr..

[19] Brown M.A., Stevenson E.J., Howatson G. (2017). Whey protein hydrolysate supplementation accelerates recovery from exercise-induced muscle damage in females. Appl. Physiol. Nutr. Metab..

[20] Clifford T., Allerton D.M., Brown M.A., Harper L., Horsburgh S., Keane K.M., Stevenson E.J., Howatson G. (2016). Minimal muscle damage after a marathon and no influence of beetroot juice on inflammation and recovery. Appl. Physiol. Nutr. Metab..

[21] Rankin P., Stevenson E., Cockburn E. (2015). The effect of milk on the attenuation of exercise-induced muscle damage in males and females. Eur. J. Appl. Physiol..

[22] Clifford T., Bell O., West D.J., Howatson G., Stevenson E.J. (2016). The effects of beetroot juice supplementation on indices of muscle damage following eccentric exercise. Eur. J. Appl. Physiol..

[23] Clifford T., Howatson G., West D.J., Stevenson E.J. (2017). Beetroot juice is more beneficial than sodium nitrate for attenuating muscle pain after strenuous eccentric-bias exercise. Appl. Physiol. Nutr. Metab..

[24] Snijders T., Smeets J.S., van Vliet S., van Kranenburg J., Maase K., Kies A.K., Verdijk L.B., van Loon L.J.C. (2015). Protein Ingestion before Sleep Increases Muscle Mass and Strength Gains during Prolonged Resistance-Type Exercise Training in Healthy Young Men. J. Nutr..

[25] Shearer D.A., Sparkes W., Northeast J., Cunningham D.J., Cook C.J., Kilduff L.P. (2017). Measuring recovery: An adapted Brief Assessment of Mood (BAM+) compared to biochemical and power output alterations. J. Sci. Med. Sport.

[26] Trommelen J., Holwerda A.M., Kouw I.W., Langer H., Halson S.L., Verdijk L.B., van Loon L.J. (2016). Resistance exercise augments postprandial overnight muscle protein synthesis rates. Med. Sci Sports Exerc..

[27] Burd N.A., Tang J.E., Moore D.R., Phillips S.M. (2009). Exercise training and protein metabolism: Influences of contraction, protein intake, and sex-based differences. J. Appl. Physiol..

[28] Warren G.L., Lowe D.A., Armstrong R.B. (1999). Measurement tools used in the study of eccentric contraction-induced injury. Sports Med..

